# Introducing an index on prediction of post-revascularization cerebral infarction using preoperative CT perfusion parameters in moyamoya disease

**DOI:** 10.1186/s13244-024-01882-7

**Published:** 2025-01-02

**Authors:** Xiaojun Hao, Chao Zhang, Chen Yang, Xintong Zhao, Yunfeng Zhou, Juan Wang

**Affiliations:** 1 Department of Radiology, The First Affiliated Hospital of Wannan Medical College, Wuhu, PR China; 2 Department of Neurosurgery, The First Affiliated Hospital of Wannan Medical College, Wuhu, PR China

**Keywords:** Moyamoya disease, Cerebral revascularization, Cerebral infarction, Perfusion imaging, Tomography (X-ray computed)

## Abstract

**Objective:**

To determine the value of preoperative CT perfusion (CTP) parameters for prediction of post-revascularization cerebral infarction (post-CI) in adults with moyamoya disease (MMD).

**Methods:**

This retrospective study included 92 adults with MMD who underwent surgical revascularization. Preoperative quantitative CTP parameters, including cerebral blood flow (CBF), cerebral blood volume (CBV), mean transit time (MTT), time to drain (TTD), and transit time to maximum of the residue function (Tmax), along with clinical data, were compared between the groups with and without post-CI. Predictors of post-CI were identified and assessed using multivariable logistic regression and receiver-operating characteristic curve analyses.

**Results:**

Post-CI occurred in 11 patients (12.0%). In univariate analysis, preoperative mean values for CBF, MTT, TTD, Tmax, initial presentation, infarction within the 2 months before surgery, surgical side, and modified Rankin Scale score on admission were associated with post-CI (all *p* < 0.05). Multivariable logistic regression revealed that the preoperative mean Tmax (OR 2.342, 95% CI: 1.267–4.330, *p* = 0.007) and infarction within the 2 months before surgery (OR 14.345, 95% CI: 2.108–97.638, *p* = 0.006) were independent predictors of post-CI. The preoperative mean Tmax produced the largest area under the curve (0.955, 95% CI: 0.914–0.997) with a cutoff of 3.590 s (sensitivity, 100%; specificity, 87.7%).

**Conclusions:**

Adults with MMD are at risk of post-CI when the preoperative mean Tmax is > 3.590 s. Cerebral infarction during the 2 months before revascularization is also a risk factor for post-CI.

**Critical relevance statement:**

Post-CI is a serious complication for adults with MMD following surgical revascularization. The risk of post-CI can be predicted using preoperative CTP parameters, which will assist neurosurgeons with surgical decisions and implementing individualized prophylactic strategies.

**Key Points:**

- Predicting the risk of post-CI in MMD patients is beneficial to their prognosis.
- The preoperative mean Tmax was an excellent perfusion parameter for predicting post-CI.
- Preoperative CTP evaluation can help clinicians make cautious surgical decisions.

**Graphical Abstract:**

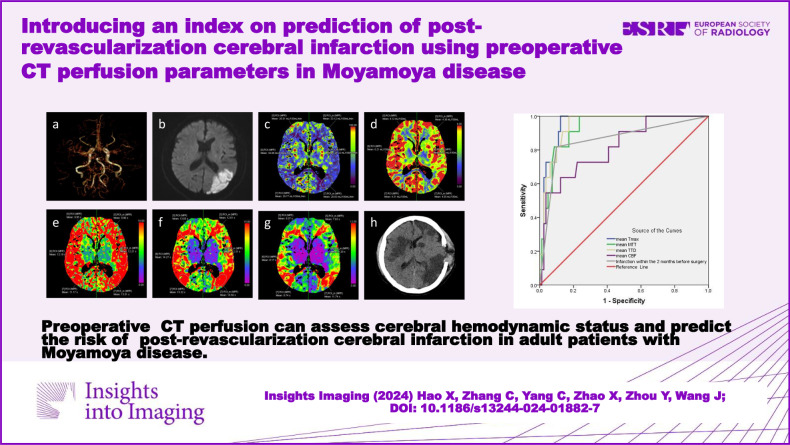

## Introduction

Moyamoya disease (MMD) is a chronic cerebrovascular disease characterized by progressive steno-occlusion of the internal carotid terminus accompanied by the formation of abnormal vascular networks, which can result in ischemic or hemorrhagic stroke [1]. At present, there are no drugs that can halt the progression of MMD. Surgical revascularization, including direct bypass, indirect bypass, and combined bypass, is generally recognized as an effective strategy that improves cerebral hemodynamics in these patients and reduces their risk of further strokes [2–4]. However, various complications had been observed after surgical revascularization, including post-revascularization cerebral infarction (post-CI), hyperperfusion syndrome, intracranial hemorrhage, subdural or epidural hematoma, and epilepsy [5–8]. Post-CI is both a common postoperative complication and a major cause of disability and irreversible neurological dysfunction. The incidence of post-CI ranges from 3.5% to 25.6% [9–13]. Therefore, early and accurate prediction of the risk of post-CI as part of preoperative management is critical to improving the outcome in patients with MMD. Previously, efforts to identify risk factors for post-CI have focused mainly on clinical and cerebrovascular characteristics [5, 9–13], such as preoperative presentation, Suzuki stage, and posterior cerebral artery involvement, with few including analyses of preoperative perfusion parameters. However, evaluation of cerebral perfusion is necessary for the determination of baseline cerebral hemodynamic status and surgical decision-making for patients with MMD [14].

Computed tomography perfusion (CTP) is an imaging technique for preoperative evaluation and postoperative follow-up of patients with MMD that can provide important information on various functional parameters relating to tissue perfusion and circulatory status [15]. However, no method for risk assessment using preoperative CTP to predict post-CI has been established, and it is unknown which parameters can be utilized. Therefore, this study investigated the value of preoperative CTP parameters in the prediction of post-CI following revascularization surgery in adult patients with MMD.

## Methods

### Study population

Medical data for adult patients with MMD between October 2016 and April 2024 were retrospectively reviewed. The study protocol was approved by our institutional review board, and the need for informed consent was waived in view of the observational nature of the research. The inclusion criteria were as follows: age ≥ 18 years; MMD confirmed by digital subtraction angiography according to the guidelines published in 2022 [16]; and CTP performed within 1 week before surgical revascularization. Patients with poor image quality or incomplete imaging data were excluded.

### Clinical characteristics and radiological findings

Baseline clinical data were collected, including age, sex, medical history (e.g., hypertension and diabetes), initial presentation (transient ischemic attack, infarction, hemorrhage, or other), infarction within the 2 months before surgery, the modified Rankin scale (mRS) score on admission, and side and type of surgery. Radiological findings included preoperative CTP parameters, posterior cerebral artery involvement, and Suzuki stage based on digital subtraction angiography. The mRS score and Suzuki stage were assessed with reference to previous reports [17, 18].

Post-CI was defined as newly developed symptomatic or asymptomatic ischemic cerebral infarction within 1 week after surgery that was confirmed by CT or diffusion-weighted imaging. Massive infarction was defined as post-CI involving three or more cerebral lobes, whereas involvement of one or two lobes was categorized as regional infarction.

### Surgical strategies

The superficial temporal artery-middle cerebral artery anastomosis is the most common direct bypass surgery for adults with MMD [2]. Indirect bypass was performed when there was no suitable donor or recipient vessel, including encephalo-myo-synangiosis, encephalo-dura-myo-synangiosis, and encephalo-dura-arterial-myo-synangiosis [19]. Combined bypass surgery integrates these two surgical techniques.

The patient selection criteria for surgical revascularization at our institution were as follows: Suzuki stage ≥ 2; clinical manifestations of disease-related cerebral ischemia or intracranial hemorrhage; hemodynamic damage evidenced by CTP evaluation; and exclude other surgical contraindications. The hemisphere exhibiting symptoms and significantly impaired hemodynamics was the preferred side for treatment. Combined bypass surgery was the preferred method for adults with MMD in our institution, and the anastomotic patency of the superficial temporal artery-middle cerebral artery was confirmed by intraoperative indocyanine green angiography.

### CTP imaging protocol and post-processing

One-stop whole-brain CTP was performed using dual-source CT scanners (SOMATOM Definition Flash; Siemens Healthcare, Erlangen, Germany) within one week before surgery. A non-contrast CT scan was performed first, with a tube voltage of 120 kVp and a tube current of 390 mAs. Next, each patient received 55 mL of nonionic contrast agent (Ioversol, 350 mg iodine/mL; Hengrui, Jiangsu, China), which was injected intravenously at a flow rate of 5 mL/s, followed by 40 mL of saline, which was injected at the same rate using a high-pressure syringe. After injection of the contrast material, CTP images were acquired using a tube voltage of 80 kVp, a tube current of 100 mAs, and a section collimation of 32 × 1.2 mm, with a z-flying focal spot using a 4D spiral mode to cover a 150-mm range from the base of the skull through to the apex of the head. The images were reconstructed with a section thickness of 3 mm and an increment of 3 mm using an H30f convolution kernel for perfusion analysis. Additionally, 1.5-mm thin-slice images were reconstructed for CT angiography (CTA) analysis.

All post-processing steps were performed on a Siemens workstation (Siemens syngo.via VB10B) using CT Neuro Perfusion software for perfusion. After step-by-step motion artifact correction, 4D noise reduction, tissue segmentation, and identification of arteries and veins, we generated color-coded perfusion parameter maps for cerebral blood flow (CBF), cerebral blood volume (CBV), mean transit time (MTT), time to drain (TTD), and transit time to the center of the impulse response function (Tmax). Images of the optimal period of cerebral artery filling were selected to transfer to CT NeuroVascular software for CTA analysis.

### Imaging analysis

**Fig. 1:**
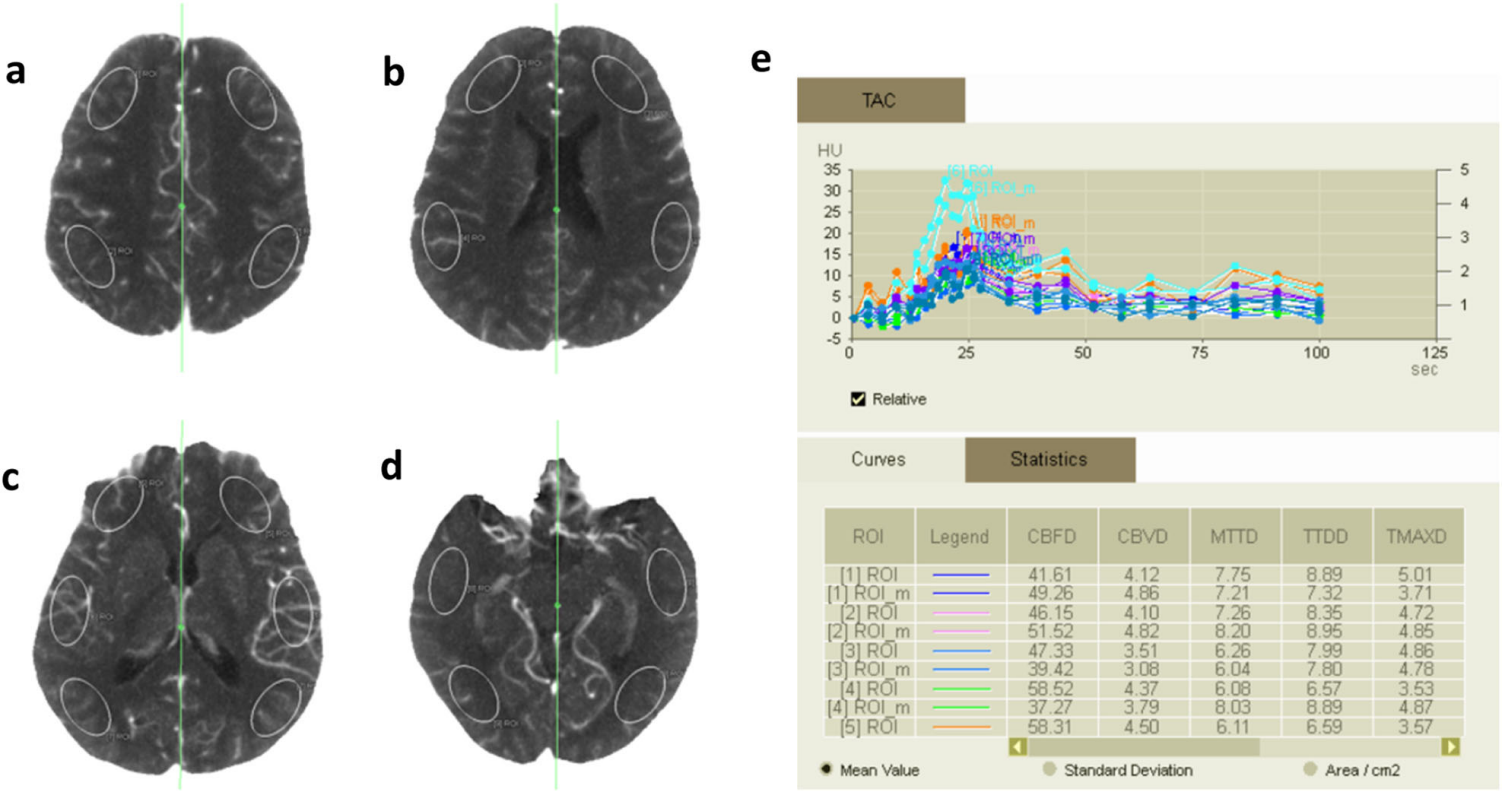
An illustration of the placement of 18 ROIs on four slices for quantitative CTP analysis. Nine ROIs were placed manually throughout the cortical flow territories on four standard levels, including the centrum semiovale (**a**), body of lateral ventricle (**b**), basal ganglia (**c**), and mesencephalon (**d**). Nine mirrored ROIs were automatically generated in the contralateral hemisphere. The quantitative values of CBF, CBV, MTT, TTD, and Tmax are automatically calculated for each ROI by the post-processing software (**e**)

For quantitative CTP analysis, a total of 18 standardized elliptical regions of interest (ROIs) were created on four slices (Fig. 1). On one side of the maximum intensity projection map for each of the four slices, 9 ROIs were placed manually along the cortical flow regions of the anterior cerebral artery, middle cerebral artery, and posterior cerebral artery, each with an area of approximately 4 cm^2^. Nine mirrored ROIs were automatically generated in the contralateral hemisphere. An attempt was made to select the same position for each measurement and avoid obvious large blood vessels, calcified areas, and malacic foci. The mean value was automatically obtained from each ROI for each of the five perfusion parameter maps. The mean values of the 18 regions of ROIs were calculated and designated as mean CBF, mean CBV, mean MTT, mean TTD, and mean Tmax. All the preoperative clinical and imaging data were analyzed by an experienced radiologist and neurosurgeon, and any disagreement was resolved by consensus.

### Statistical analysis

Continuous variables that were normally distributed are presented as the mean ± standard deviation and were compared between groups using the independent *t*-test. Continuous variables with a skewed distribution are shown as the median (interquartile range) and were compared between groups using the Mann–Whitney *U-*test. Categorical variables are presented as the frequency (percentage) and were compared between groups using the chi-squared test or Fisher’s exact test. Variables with a *p*-value < 0.05 in univariate analysis were included in multivariate logistic regression analysis to identify predictors of post-CI. Receiver-operating characteristic (ROC) curve analysis was performed to evaluate the prediction performance for post-CI, with the calculation of the area under the curve (AUC), the corresponding optimal threshold value, and sensitivity and specificity. The statistical analysis was performed using SPSS version 23.0 (IBM Corp., Armonk, NY, USA). A *p*-value < 0.05 was considered statistically significant.

## Results

**Fig. 2:**
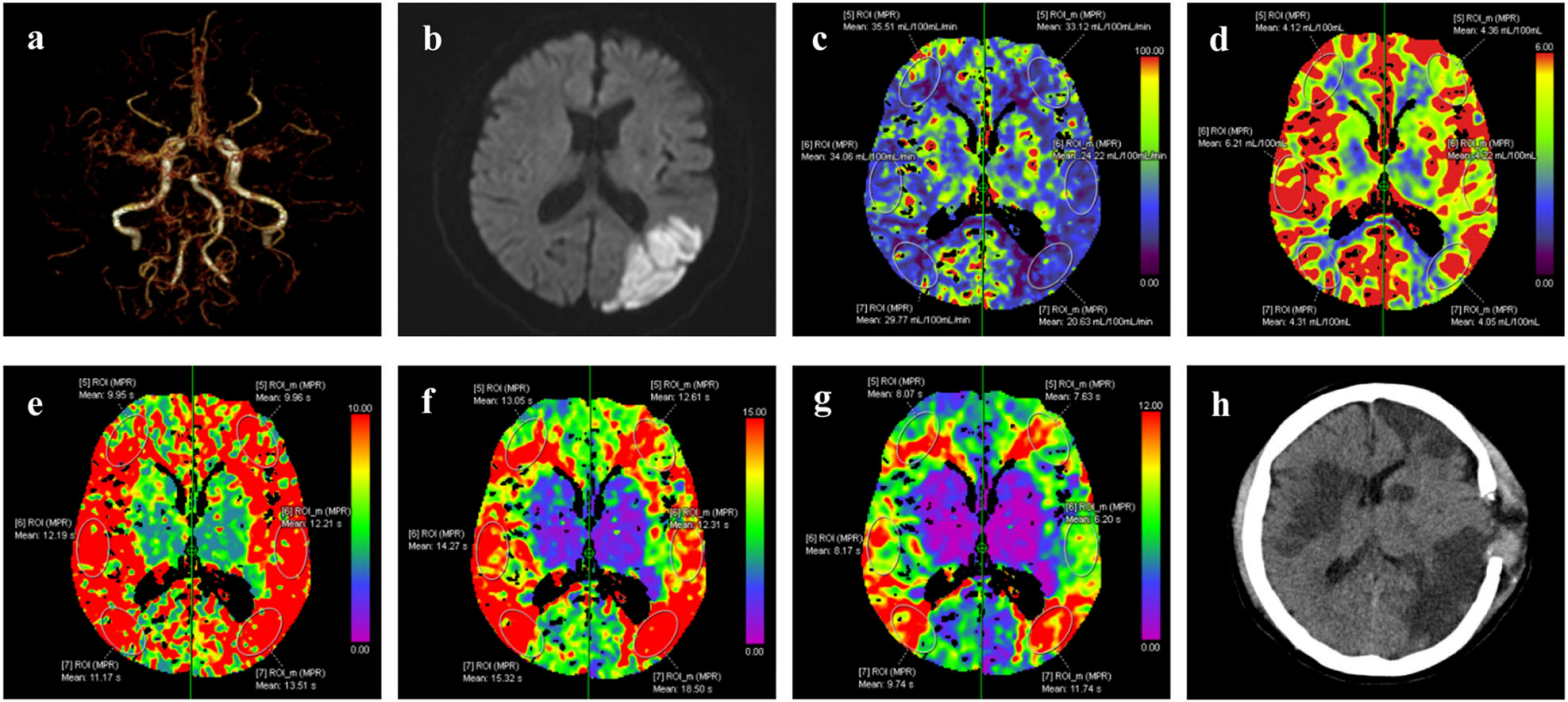
A 50-year-old male MMD patient with post-CI. Preoperative CTA showed MMD (**a**). The diffusion-weighted imaging performed two weeks before surgery demonstrated an acute cerebral infarction in the left parietal-occipital lobe (**b**). Preoperative CTP showed a significant decrease in CBF (**c**), a mild decrease in CBV (**d**), and a significant increase in MTT (**e**), TTD (**f**), and Tmax (**g**) in both hemispheres. Right hemiplegia appeared on the second day after surgery, followed by coma. Postoperative CT showed multiple newly developed cerebral infarctions in both hemispheres (**h**)

**Fig. 3:**
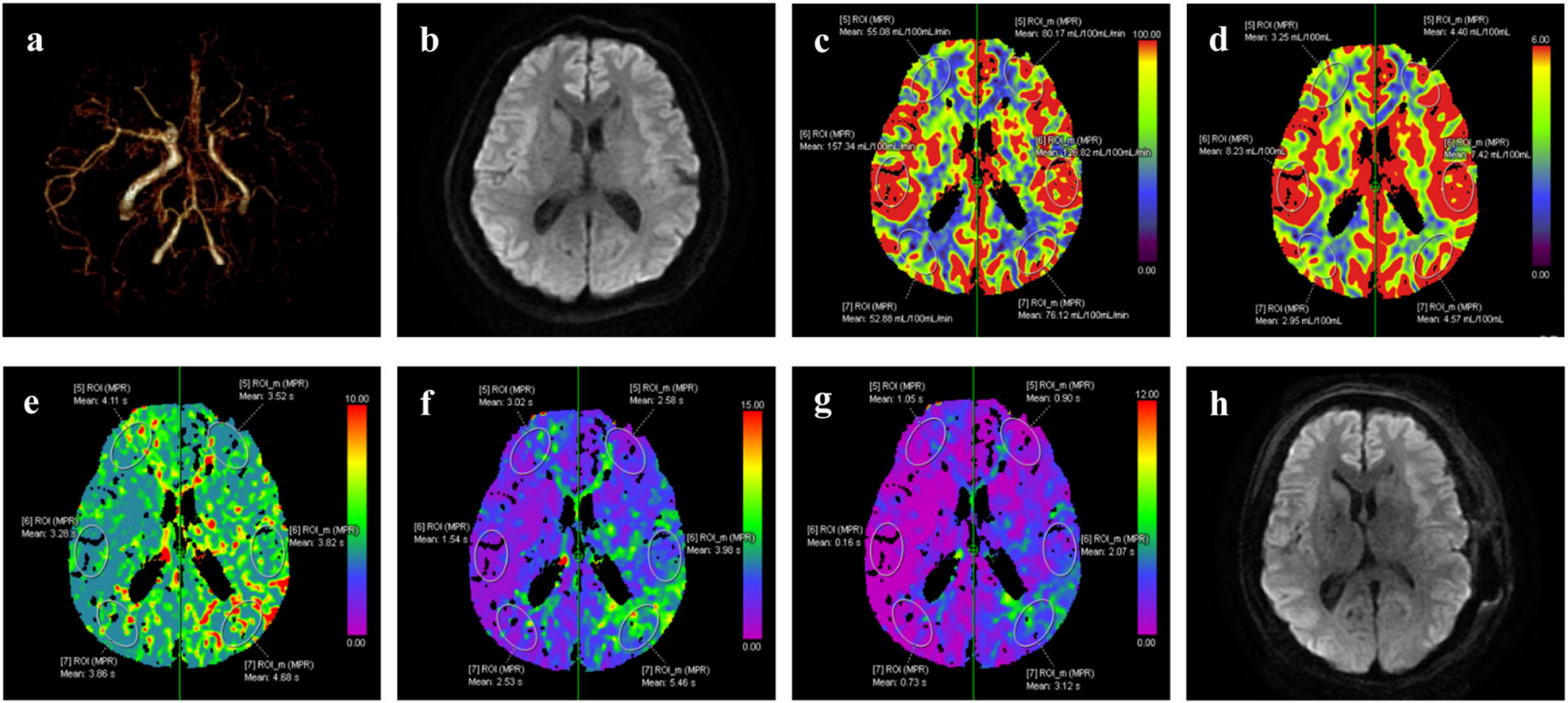
A 50-year-old male MMD patient without post-CI. Preoperative CTA showed MMD (**a**). Preoperative diffusion-weighted imaging showed no acute cerebral infarction (**b**). Preoperative CTP showed no obvious changes in CBF (**c**), CBV (**d**), and a mild increase in MTT (**e**), TTD (**f**), and Tmax (**g**) in the left temporal-parietal lobe and right frontal lobe. Postoperative diffusion-weighted imaging showed no cerebral infarction (**h**)

Ninety-two adult patients with MMD (49 women, 43 men; mean age 49.7 ± 9.2 years) were included in the study. Their clinical, demographic, and imaging data are summarized in Table 1. Eleven of the 92 patients (12%) developed post-CI, which was ipsilateral to the surgical side in six patients and bilateral in five. Post-CI was massive in four patients and regional in seven. Detailed information on the patients with post-CI is provided in Table 2. Representative images for a patient who developed post-CI and one who did not are shown in Figs. 2 and 3.

**Table 1:** Clinical, demographic, and imaging characteristics in MMD patients with and without post-CI

Characteristic	All, (*n* = 92)	Post-CI, (*n* = 11)	Non post-CI, (*n* = 81)	*p-*value
Age, years	49.7 ± 9.2	49.6 ± 6.9	49.7 ± 9.5	0.992
Female	49 (53.2)	5 (45.5)	44 (54.3)	0.580
Hypertension	39 (42.4)	7 (63.6)	32 (39.5)	0.232
Diabetes	13 (14.1)	3 (27.3)	10 (12.3)	0.383
Initial presentation				0.001*
TIA	31 (33.7)	4 (36.4)	27 (33.3)	
Infarction	20 (21.7)	7 (63.6)	13 (16.1)	
Hemorrhage	35 (38.1)	0 (0)	35 (43.2)	
Others	6 (6.5)	0 (0)	6 (7.4)	
Infarction within the 2 months before surgery	17 (18.5)	9 (81.8)	8 (9.9)	< 0.001*
Suzuki stage				0.392
2	2 (2.2)	0 (0)	2 (2.5)	
3	53 (57.6)	7 (63.6)	46 (56.8)	
4	30 (32.6)	2 (18.2)	28 (34.5)	
5	7 (7.6)	2 (18.2)	5 (6.2)	
Surgery side				0.021*
Left	52 (56.5)	10 (90.9)	42 (51.9)	
Right	40 (43.5)	1 (9.1)	39 (48.1)	
Surgery type				0.201
Combined bypass	77 (83.7)	11 (100)	66 (81.5)	
Indirect bypass	15 (16.3)	0 (0)	15 (18.5)	
mRS score on admission				
Mean	2.25 ± 1.05	2.91 ± 0.70	2.16 ± 1.01	0.007*
0–2	52 (56.5)	3 (27.3)	49 (60.5)	0.052
3–5	40 (43.5)	8 (72.7)	32 (39.5)	
PCA involvement	35 (38.0)	5 (45.5)	30 (37.0)	0.742
Preoperative quantitative CTP				
Mean CBV, mL min^−^^1^	4.03 ± 0.48	4.10 ± 0.61	4.04 ± 0.47	0.635
Mean CBF, mL.100 g^−^^1^ min^−^^1^	56.71 (51.71, 62.66)	45.19 (37.90, 55.76)	57.43 (52.16, 63.70)	0.001*
Mean MTT, s	5.24 (4.66, 6.12)	6.87 (6.53, 8.56)	5.11 (4.61, 5.67)	< 0.001*
Mean TTD, s	5.25 (4.43, 6.67)	8.40 (7.15, 10.54)	5.06 (4.27, 6.07)	< 0.001*
Mean Tmax, s	2.63 (2.08, 3.54)	4.96 (3.92, 6.26)	2.51 (1.86, 3.28)	< 0.001*

*MMD* moyamoya disease, *Post-CI* post-revascularization cerebral infarction, *TIA* transient ischemic attack, *mRS* modified Rankin scale, *PCA* posterior cerebral artery, *CTP* computed tomography perfusion, *CBF* cerebral blood flow, *CBV* cerebral blood volume, *MTT* mean transit time, *TTD* time to drain, *Tmax* transit time to the center of the impulse response function

* *p* < 0.05

**Table 2:** Detailed information on the patients with post-CI

No	Age/sex	Initial presentation	Suzuki stage	Infarction within the 2 months before surgery	Surgery side	Mean Tmax, (s)	Onset (POD)	Characteristics of post-CI			AdmissionmRS	DischargemRS
								Location	Size	Symptom		
1	34/F	Infarction	3	Yes	Left	4.97	2	Bilateral	Massive	Hemiplegia	3	5
2	46/F	Infarction	3	Yes	Left	3.61	2	Ipsilateral	Regional	Hemiplegia	2	4
3	49/M	Infarction	4	Yes	Left	4.93	4	Bilateral	Massive	Hemiplegia	3	4
4	45/M	TIA	3	Yes	Left	3.83	3	Bilateral	Regional	Weakness	2	3
5	46/M	Infarction	3	Yes	Right	6.22	2	Ipsilateral	Massive	Coma	3	5
6	55/M	Infarction	3	Yes	Left	8.55	5	Ipsilateral	Regional	Epilepsy	3	3
7	58/F	Infarction	5	Yes	Left	6.26	5	Bilateral	Massive	Coma	4	5
8	52/F	TIA	3	No	Left	4.96	3	Ipsilateral	Regional	Aphasia	2	4
9	57/F	TIA	3	No	Left	3.92	7	Ipsilateral	Regional	Aphasia	3	3
10	54/M	Infarction	4	Yes	Left	4.60	5	Ipsilateral	Regional	None	4	4
11	50/M	TIA	5	Yes	Left	8.73	2	Bilateral	Massive	Coma	3	5

*post-CI* post-revascularization cerebral infarction, *TIA* transient ischemic attack, *Tmax* transit time to the center of the impulse response function, *POD* postoperative day, *mRS* modified Rankin scale

Univariate analysis revealed significant associations of initial presentation (*p* = 0.001), infarction within the 2 months before surgery (*p* < 0.001), surgical side (*p* = 0.021), and mRS score on admission (*p* = 0.007) with post-CI. The preoperative quantitative CTP parameters of mean MTT, mean TTD, and mean Tmax was significantly higher (all *p* < 0.001), while mean CBF (*p* = 0.001) was significantly lower in the post-CI group than those in the non-post-CI group, with no significant difference in preoperative mean CBV. Multivariable logistic regression analysis showed that the preoperative mean Tmax (OR 2.342, 95% CI: 1.267–4.330, *p* = 0.007) and infarction within the 2 months before surgery (OR 14.345, 95% CI: 2.108–97.638, *p* = 0.006) were significant independent predictors of post-CI.

**Fig. 4:**
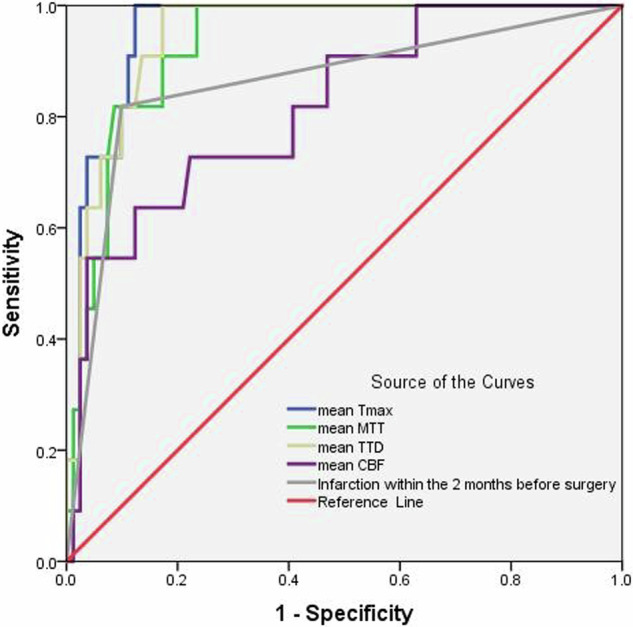
ROC curves prediction post-CI based on preoperative CTP parameters and clinical data. The mean Tmax produced the largest AUC with a cutoff of 3.590 s (sensitivity, 100%; specificity, 87.7%)

The ROC for the above-mentioned independent predictors revealed that the preoperative mean Tmax had the largest AUC (0.955, 95% CI: 0.914–0.997) with a cutoff value of 3.590 s (sensitivity, 100%; specificity, 87.7%) for prediction of post-CI in adult patients with MMD (Fig. 4).

## Discussion

Revascularization surgery continues to be the primary treatment for patients with MMD. However, post-CI remains a serious complication that needs to be overcome. Early identification of patients with MMD at risk of developing post-CI by preoperative evaluation is desirable for the implementation of an individualized prophylactic or prudent surgical strategy. The present study focused mainly on preoperative CTP parameters and their role in the prediction of post-CI in adults with MMD. The results identified a higher preoperative mean Tmax as a better predictor of post-CI. Furthermore, patients with MMD who had experienced cerebral infarction within the 2 months before revascularization surgery had an increased likelihood of developing post-CI.

The precise mechanism underlying the development of post-CI in patients with MMD is not clearly understood and is likely to be multifactorial. Previous studies have shown that preoperative impairment of cerebrovascular reserve and hemodynamic instability are the primary causes of post-CI [20–22], which is consistent with our present research findings. The CTP results of this study indicated that patients with MMD who develop post-CI have severe cerebral perfusion deficits before surgery. Quantitative and qualitative CTP parameters can be used to assess cerebral perfusion status, enabling the prediction of ischemic complications in various scenarios, such as aneurysmal subarachnoid hemorrhage and MMD [23–25]. During the compensatory stage of MMD, CBF is normally maintained by elevated CBV to supply brain tissue. As the disease progresses further, the self-expanding regulation of small vessels is insufficient to maintain CBF, resulting in a state of cerebral hypoperfusion, which is manifested by a decrease in CBF and an increase in MTT, TTD, and Tmax [23]. The increase in MTT reflects a decrease in capillary perfusion pressure, and the increase in TTD reflects a prolonged outflow time of the contrast agent. The time-related CTP parameters (MTT and TTD) are more sensitive for detecting hemodynamic changes than CBF and CBV [25]. In recent years, Tmax has been considered a promising parameter to assess the prognosis of patients with ischemic stroke [26]. In our study, the preoperative mean Tmax emerged as a significant independent predictor of post-CI, producing the largest AUC (0.955) and a cutoff value of 3.590 s with 100% sensitivity and 87.7% specificity. A recent study also identified Tmax to be a useful parameter that was correlated with the clinical outcome in adult patients with MMD, reporting that the significant predictive factor for post-CI was a larger volume of preoperative Tmax > 6 s and the cutoff value was 59.5 mL [27]. Tmax represents the time point at which blood storage in brain tissue reaches its maximum value and is a sensitive indicator of blood flow reserve, collateral circulation status, and microcirculatory perfusion of the brain [28]. Compared with the other CTP parameters, Tmax is more affected by delayed arrival of blood flow through indirect macrovascular pathways [29]. A higher Tmax may indicate poor collateral circulation and impaired self-regulation of the cerebrovascular microcirculation. Therefore, we speculate that patients with MMD who have a higher mean Tmax may be less able to withstand the trauma and hemodynamic changes caused by surgery and be at an increased risk of postoperative hypoperfusion of brain tissue and cerebral infarction.

Early surgical intervention often leads to better long-term clinical outcomes in patients with MMD, as the disease itself has a progressive natural course. However, for patients with MMD in the acute infarction phase, immediate surgery can lead to serious complications. An increasing number of studies have identified preoperative cerebral infarction to be a risk factor for post-CI in patients with MMD [5, 9, 21, 22, 30], even regardless of infarct size and location [22]. Chen et al found a significant association of postoperative cerebral ischemia with an interval of less than 8 weeks between transient ischemic attack or presentation with infarction and surgery [13]. This study found a similar result that patients with MMD who initially presented with infarction, especially those who experienced infarction within the 2 months before revascularization surgery, were more likely to develop post-CI. Several studies have explored the effect of the timing of surgery following acute cerebral infarction on the risk of post-CI in patients with MMD. Kim et al recommended that revascularization surgery be delayed for at least 6 weeks after the most recent cerebral infarction in pediatric patients with ischemic MMD [31]. Other researchers found that patients with MMD who underwent surgical revascularization more than 90 days after acute infarction had fewer postoperative ischemic complications in comparison with their counterparts who underwent early surgery, with no significant increase in the risk of a further stroke or worsening disease manifestations [32]. One possible explanation for this finding may be that the cerebral hemodynamic state is more unstable during the early stages after infarction in patients with MMD. Such patients may be more susceptible to being disturbed by surgery because they have not yet recovered from the attack of the latest acute onset, as time goes on, the body’s self-regulation gradually stabilizes the cerebral hemodynamic state. Therefore, for patients with MMD who experienced infarction within the 2 months before surgery, more caution is required when deciding to undergo revascularization and perioperative management.

In addition, the surgery side and mRS score on admission were also associated with post-CI in this study, which is consistent with previous reports [33, 34]. Post-CI occurred more prevalent in patients who underwent left-sided surgery, which might be due to the predominance of the left hemisphere in language processing, making left-sided cerebral infarctions more easily recognized [35]. Meanwhile, patients with higher mRS scores on admission may have recently experienced events that resulted in neurological deficits, and their cerebrovascular self-regulation might still be in a relatively unstable state.

This study has several limitations. First, it had a single-center retrospective design and a relatively small sample size, which could have led to selection bias. Second, it did not evaluate perfusion in the cerebellar hemisphere and brainstem, where skull base artifacts could easily affect the accuracy of perfusion measurements. Third, the ischemic and hemorrhagic MMD may have different underlying pathophysiological mechanisms, we did not perform a subgroup analysis for these two groups. Finally, the algorithm for CTP varies in different vendors, and the exact value of Tmax will also vary.

## Conclusions

Preoperative whole-brain CTP can assess cerebral perfusion status and predict the risk of post-CI after revascularization surgery in adult patients with MMD. When the preoperative mean Tmax value is greater than 3.590 s, a patient with MMD may be at increased risk of post-CI. The risk of post-CI is also higher if a patient with MMD has experienced a cerebral infarction within the 2 months before surgery.

## Data Availability

The datasets used for analyses during the current study are available from the corresponding authors upon reasonable request.

## Abbreviations

AUC: Area under the curve
CBF: Cerebral blood flow
CBV: Cerebral blood volume
CTA: Computed tomography angiography
CTP: Computed tomography perfusion
MMD: Moyamoya disease
mRS: modified Rankin scale
MTT: Mean transit time
Post-CI: Post-revascularization cerebral infarction
ROC: Receiver-operating characteristic
ROI: Region of interest
Tmax: Transit time to the center of the impulse response function
TTD: Time to drain
